# Phase II trial of dose-dense chemotherapy followed by dose-intense erlotinib for patients with newly diagnosed metastatic non-small cell lung cancer

**DOI:** 10.3892/ijo.2013.2122

**Published:** 2013-10-03

**Authors:** W. JEFFREY PETTY, JENNIFER LAUDADIO, LYNSAY BRAUTNICK, JAMES LOVATO, TRAVIS DOTSON, NATHAN P. STREER, KATHRYN E. WEAVER, ANTONIUS A. MILLER

**Affiliations:** 1 Departments of Medicine, Section on Hematology and Oncology, Wake Forest University, Wake Forest School of Medicine, Winston-Salem, NC 27157, USA; 2 Cancer Biology, Wake Forest University, Wake Forest School of Medicine, Winston-Salem, NC 27157, USA; 3 Pathology, Wake Forest University, Wake Forest School of Medicine, Winston-Salem, NC 27157, USA; 4 Biostatistical Sciences, Wake Forest University, Wake Forest School of Medicine, Winston-Salem, NC 27157, USA; 5 Medicine, Section on Pulmonary Medicine, Wake Forest University, Wake Forest School of Medicine, Winston-Salem, NC 27157, USA; 6 Social Sciences and Health Policy, Wake Forest University, Wake Forest School of Medicine, Winston-Salem, NC 27157, USA; 7 Comprehensive Cancer Center of Wake Forest University, Wake Forest School of Medicine, Winston-Salem, NC 27157, USA

**Keywords:** erlotinib, pharmacokinetics, cyclin D1, cyclin D3

## Abstract

This phase II study investigated dose-intense erlotinib maintenance after dose-dense chemotherapy for patients with metastatic non-small cell lung cancer and examined two cell cycle biomarkers. Patients with newly diagnosed metastatic non-small cell lung cancer received docetaxel 75 mg/m^2^ and cisplatin 75 mg/m^2^ on day 1 and pegfilgrastim on day 2 every 14 days for four cycles. Patients then received erlotinib with initial doses based on smoking status. Doses were increased in 75 mg increments every two weeks depending on toxicities until each patient's maximal tolerable dose (MTD) was achieved. Cyclin D1 and D3 biomarkers were measured by immunohistochemistry. The objectives of the study were to evaluate time to progression (TTP) and overall survival (OS) for the entire population and biomarker subgroups. Forty-five patients were enrolled. Intra-patient erlotinib MTD ranged from 0 to 525 mg. Median MTD achieved in smokers was higher than in non-smokers (300 vs. 150 mg; P=0.019). TTP for the entire cohort was not significantly improved compared to historical controls. Patients with high cyclin D1 expressing tumors demonstrated improved TTP on erlotinib (8.2 vs. 4.7 months; hazard ratio, 4.1; 95% CI, 1.6–0.6; P=0.003) and improved OS (20.5 vs. 8.0 months; hazard ratio 2.8; 95% CI, 1.2–6.3; P=0.016). Intratumoral cyclin D3 expression did not impact clinical outcomes. Current smokers but not former smokers exhibit a higher erlotinib MTD. High cyclin D1 expression was associated with favorable TTP and OS.

## Introduction

Dose-dense docetaxel and cisplatin was previously investigated as a first line treatment for patients with metastatic non-small cell lung cancer (1). The toxicities included significant neuropathy, nausea and dehydration. The response rate achieved with this regimen was 53% and the overall survival was 11 months with a 1-year survival rate of 45%. Maintenance treatment was not given following completion of dose-dense chemotherapy in this prior study.

Erlotinib improves the overall survival of patients with metastatic non-small cell lung cancer in the maintenance, second line and third line settings (2,3). The improvement in overall survival is longer for patients who achieve a rash after starting treatment with erlotinib (4,5). Patients who continue to smoke cigarettes are less likely to experience rash and are less likely to benefit from treatment. These observations suggest that a failure to achieve adequate drug levels may contribute to clinical erlotinib resistance in the population of patients who continue to smoke cigarettes.

A phase I/II study investigated the maximal tolerable dose (MTD) of erlotinib in patients who were smoking ≥10 cigarettes daily (6). This study found that the dose of 300 mg daily in smokers achieved a similar pharmacokinetic and side effect profile to non-smoking patients treated with 150 mg daily. The increase in erlotinib metabolism was attributed to induction of CYP1A1/1A2 enzymes by exposure to tobacco smoke. However, this prior phase I/II study did not examine the MTD of former smokers.

Molecular genetic properties of individual cancers such as epidermal growth factor receptor (EGFR) mutations impact the likelihood of achieving clinical benefit with treatment. Progression-free survival is dramatically increased in patients with certain EGFR activating mutations (3,7–9). Patients with EGFR wild-type cancers treated with erlotinib also experience improved progression-free survival and overall survival in the maintenance setting (3) and additional biomarkers are needed for clinical decision making.

Erlotinib functions by inducing cell cycle arrest at the G1 checkpoint (10). Cell cycle arrest is triggered by the transcriptional repression of the cyclin D1 cell cycle regulatory protein (11). This effect has been documented in both EGFR mutant and erlotinib-sensitive, EGFR wild-type lung cancer cell lines (11,12). Erlotinib-resistant, EGFR wild-type lung cancers do not exhibit this effect (11). The combination of bexarotene and erlotinib also has been shown to reduce cyclin D1 expression (13) and the BATTLE trial found that high intratumoral cyclin D1 predicted favorable clinical outcomes with this combination (14). *In vitro* studies have shown that cyclin D3 is not repressed by erlotinib treatment and that high cyclin D3 expression is associated with erlotinib resistance (15). Based on this prior study, we hypothesized that high cyclin D1 expression would predict favorable outcomes and high cyclin D3 expression would predict unfavorable outcomes with dose-intense erlotinib maintenance.

## Materials and methods

### Eligibility

Patients were required to have stage IV non-small cell lung cancer. All patients were required to have a documented histopathologic or cytopathologic diagnosis. Patients were allowed to have either measurable disease or evaluable disease. ECOG performance status (PS) of 0 or 1 was required. Patients were ineligible if they had received prior chemotherapy, had inadequate organ function, were pregnant or breast feeding, or were currently receiving radiation therapy.

### Treatment plan

This study was approved by the Institutional Review Board of Wake Forest University and was registered with ClinicalTrials.gov (NCT00723138). After obtaining written informed consent, patients were treated with cisplatin 75 mg/m^2^ and docetaxel 75 mg/m^2^ with both drugs given intravenously on day 1 every two weeks. Prophylactic anti-emetics were given based on investigator's preference and generally included fosaprepitant or aprepitant as well as 5-HT3 antangonists. Prophylactic growth factor support with pegfilgrastim was administered day 2 of every cycle. Treatment was repeated for four cycles or until unacceptable toxicity or disease progression.

Erlotinib was started immediately after completion or discontinuation of chemotherapy for all patients regardless of response or progression on chemotherapy. The starting doses of erlotinib were 300 mg daily for patients who were smoking at ≥10 cigarettes per day and 150 mg daily for all other patients. Doses of erlotinib were increased in 75 mg increments every two weeks until patients developed either grade 2 or 3 toxicities (according to the National Cancer Institute Common Terminology Criteria for Adverse Events version 3.0). In the event of grade 3 toxicities, erlotinib was held until resolution to grade 1 and then the dose was reduced by 75 mg daily. If the reduced dose was tolerated with grade 2 or less toxicity, that was determined to be the MTD for that patient. In the event of grade 2 toxicities, medical interventions were added and erlotinib was continued at that dose which was determined to be the MTD for that patient.

### Study procedures

At the time of enrollment, all patients completed a detailed smoking history questionnaire. Patients were categorized as never smokers (<100 lifetime cigarettes), distant former (>1 year since cessation), former (1 year - 1 month since cessation), recent former (<1 month since cessation), and current smokers. Physical examination and standard laboratory tests were performed prior to each cycle of chemotherapy. Complete blood counts were performed weekly during dose-dense chemotherapy. After initiation of erlotinib, physical examination and laboratory tests were performed every two weeks until the patient's erlotinib MTD was established and then every four weeks. Tumor measurements were performed prior to initiation of treatment, after completion of dose-dense chemotherapy and then every eight weeks until disease progression or unacceptable toxicity. Tumor response was assessed using the Response Evaluation Criteria in Solid Tumors (RECIST) (16).

### Immunohistochemistry procedure

Formalin-fixed paraffin-embedded biopsy specimens that had been obtained prior to enrollment were analyzed after the completion of the study by a pathologist who was unaware of clinical outcomes (Jennifer Laudadio). Antigen retrieval was performed using the Leica antigen retrieval system according to the manufacturer's protocol for 20 min prior to applying antibodies (Leica Microsystems, Wetzlar, Germany). Cyclin D1 primary antibody (Clone SP4, ThermoScientific, Waltham, MA, USA) at a dilution of 1:50 and cyclin D3 primary antibody (DCS-22, Leica Microsystems) at a dilution of 1:20 were independently applied to prepared slides. The percent of cancer cells staining positive for each cyclin was determined. Tumors were then categorized as having high or low expression based on whether the expression was above or below the median percent staining result for each marker.

### EGFR mutation analyses

Genomic DNA was extracted from paraffin-embedded biopsy tissues using the DNEasy Tissue kit (Qiagen, Valencia, CA, USA) according to the manufacturer's protocol. DNA concentrations were measured using spectroscopy. Polymerase chain reaction (PCR) assays were performed using the EGFR PCR Kit Using Scorpions and Amplification Refractory Mutation System (Qiagen) to assess for 28 activating mutations in the EGFR gene. Activating mutations identified with this method were confirmed and tested for the T790M resistance mutation by a second independent analysis using Rotor Gene analysis with the EGFR RGQ PCR kit (Qiagen).

### Statistical methods

A sample size of 45 evaluable patients was selected to provide 85% power to detect an improvement in time to progression (TTP) by 2.5 months over the historical control of 4 months (17) using a two-sided test, assuming exponential distribution of times and a Type I error rate = 5%. Overall survival (OS), TTP and toxicity statistics were performed on an intent-to-treat basis. Comparisons of patient characteristics were performed using χ^2^ test except for age which was compared using t-test. OS and TTP were assessed using the Kaplan-Meier method. Comparisons of survival curves were performed using the log-rank test. Cox proportional hazards models were used to assess differences between biomarker expression groups after controlling for chemotherapy response, age and PS. All P-values shown are two-sided.

## Results

### Patients

Forty-five patients were enrolled from August, 2007 to February, 2011. The patient characteristics are displayed in Table I. All patients were eligible and received at least one cycle of chemotherapy. Five patients did not receive erlotinib for the following reasons: one died from a pulmonary embolism during dose-dense chemotherapy, two initiated treatments other than erlotinib after dose-dense chemotherapy and two discontinued all treatment. Two patients initiated erlotinib but progressed prior to achieving MTD.

### Toxicity

The toxicities during dose-dense chemotherapy and dose-intense erlotinib are displayed in Table II. Toxicities during dose-dense chemotherapy were primarily non-hematologic. Only one case of febrile neutropenia occurred. Significant fatigue, anorexia and dehydration were common, and 69% of patients received all four cycles of chemotherapy.

Toxicities during dose-intense erlotinib were primarily rash and diarrhea. However, other toxicities including anorexia, dehydration and fatigue were dose limiting in 14% of patients. No patient on protocol developed grade 5 toxicity.

### Impact of smoking on erlotinib MTD

**Figure 1.**
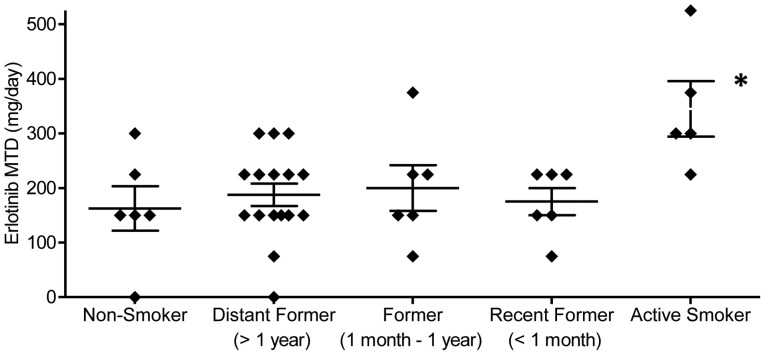
Impact of smoking status on the maximal tolerable dose (MTD) of erlotinib. MTD achieved for each patient is shown. Horizontal lines indicate mean values and standard error bars are shown. *Significant difference (P<0.05) in MTD as compared to non-smokers.

The median erlotinib MTDs for lifelong non-smokers, former smokers, and current smokers were 150, 187.5 and 300 mg daily. The MTD for current smokers was significantly higher as compared to lifelong non-smokers (P=0.019) and former smokers (P<0.001). The MTD for former smokers was not significantly different as compared to lifelong non-smokers (P=0.51). Fig. 1 depicts the MTD grouped by smoking status. Within the group of former smokers, recent (quit <1 month prior) and distant (quit >1 year prior) former smokers exhibited a similar median MTD.

### Biomarker analyses

Thirty-four patients had adequate tissue for immunohistochemical biomarker and EGFR mutation studies. Cyclin D1 expression ranged from 1 to 95% with a median of 33%. Cyclin D3 expression ranged from 5 to 85% with a median of 28%.

Samples from three patients were initially identified as potentially harboring EGFR mutations. A subsequent analysis confirmed that two of these three patients harbored EGFR activating mutations (one del 19 and one L858R). Neither of these co-expressed the T790M resistance mutation. The biomarker and clinical outcomes for these patients are shown in Table III.

### Response and survival

**Figure 2.**
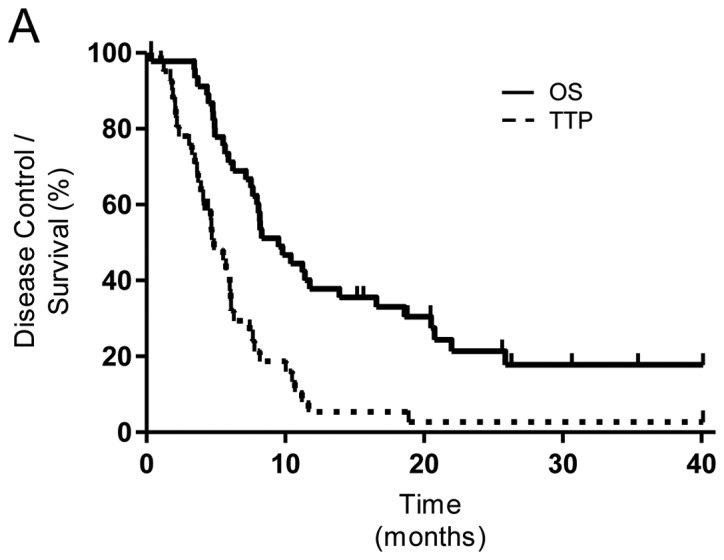

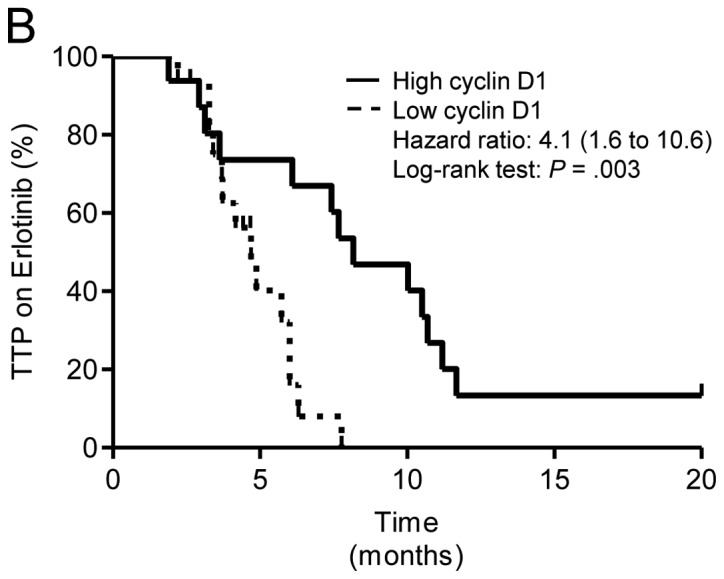

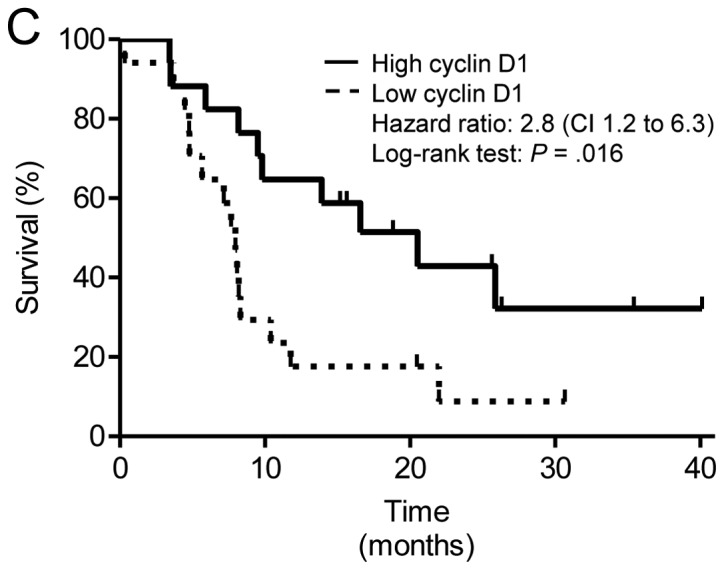
Kaplan-Meier graph and log-rank statistics based on biomarker expression. (A) Time to progression (TTP) and overall survival (OS) for all patients. (B) TTP on erlotinib for patients based on cyclin D1 expression. (C) OS for patients based on cyclin D1 expression.

Radiographic responses following dose-dense chemotherapy were partial response (20%), stable disease (63%) and progressive disease (18%). For the entire study population, the median time to progression was 4.6 months (95% CI, 3.7–6.1 months) which was not significantly improved compared to the historical control of 4 months. The median overall survival for the entire study population was 9.5 months (Fig. 2).

As compared to low cyclin D1 expression, high cyclin D1 expression was associated with longer TTP on erlotinib (8.2 vs. 4.7 months; hazard ratio, 4.1; 95% CI, 1.6–10.6; P=0.003) and improved OS (20.5 vs. 8.0 months; hazard ratio 2.8; 95% CI 1.2–6.3; P=0.016) as shown in Fig. 2. Cyclin D3 has been proposed as a marker of erlotinib resistance. However, no significant difference between high cyclin D3 and low cyclin D3 was observed for TTP on erlotinib (6.1 vs. 5.7 months; hazard ratio 1.0; 95% CI, 0.43–2.6; P=0.98) or OS (11.8 vs. 8.3 months; hazard ratio 1.3; 95% CI, 0.63–2.8; P=0.46).

Cox proportional hazards models were used to control for the potential confounding effects of chemotherapy response, age and PS on clinical outcomes. After controlling for these variables, high cyclin D1 continued to predict improvements in TTP on erlotinib (hazard ratio 3.0; 95% CI, 1.32–6.78; P=0.009) and OS (hazard ratio 3.38; 95% CI, 1.28–8.94; P=0.014). After controlling for these variables, the effect of cyclin D3 continued to be non-significant for TTP on erlotinib (P=0.71) and OS (P=0.94).

Clinical characteristics of patients with high cyclin D1 and low cyclin D1 expressing cancers are compared in Table IV. Radiographic responses to dose-dense chemotherapy were similar between these groups of patients. High cyclin D1 expressing cancers were more like to be adenocarcinomas and were more likely to present with brain or soft-tissue metastases.

## Discussion

In this study, dose-dense chemotherapy was associated with high degree of treatment related toxicities and a response rate lower than that observed in a prior multi-institutional study (1). This limits interest in future studies utilizing the dose-dense cisplatin and docetaxel regimen for unselected patients. Rapid dose-escalation of erlotinib following completion of chemotherapy was safe and well-tolerated. Increasing erlotinib dose by 75 mg every two weeks effectively achieved MTD with only two patients progressing prior to reaching MTD. While TTP and OS outcomes were lower than those obtained in the SATURN study, a direct comparison cannot be made between these studies since the SATURN population was restricted to patients with clinical benefit from initial chemotherapy treatment (stable disease, partial response, or complete response) (3).

In some situations, erlotinib resistance may be related to achieving inadequate drug levels. Intensification of erlotinib dose in the maintenance setting is an attractive approach to prevent this form of resistance. Our findings confirm prior reports indicating that the MTD of erlotinib is higher in patients who continue to smoke. A randomized phase III trial is ongoing to test whether high dose erlotinib treatment will improve the clinical outcomes over standard dose erlotinib for patients who continue to smoke (18).

In the present study, the MTD for former smokers was also examined. The median MTD for former smokers was significantly lower than for current smokers and was not elevated compared to lifelong non-smokers. These findings indicate that the increase in erlotinib metabolism triggered by tobacco exposure is reversible following smoking cessation. Providing smoking cessation interventions to patients after diagnosis of lung cancer improves clinical outcomes and reducing the risk of inadequate drug levels may help to explain this effect (19,20).

EGFR mutation analysis is a valuable test for identifying highly sensitive tumors in patients who will benefit from first line erlotinib instead of chemotherapy. Consistent with this, the patient in the present study with the longest TTP and OS demonstrated an EGFR activating mutation as well as high cyclin D1 (Table III). Some patients with EGFR wild-type cancers also benefit from erlotinib treatment (3). Several emerging biomarkers that could identify the subset of erlotinib-sensitive EGFR wild-type cancers are undergoing clinical testing. These include Ras mutations, TGF-α, E-cadherin and cyclin D1 among others (14,21–23). The present study supports high cyclin D1 expression as a marker of erlotinib sensitivity. High cyclin D3 expression failed to predict erlotinib resistance in this study.

Cyclin D1 was identified as a biomarker by studying the mechanism of action of erlotinib using *in vitro* models as well as pre- and post-treatment cancer biopsies (11). Cyclin D1 has been proposed as a nodal point for EGFR signaling with multiple pathways leading from EGFR activation to induction of this cell cycle regulator. The present study found no significant difference in chemotherapy response for high cyclin D1 expressing cancers but did show significant improvements in TTP on erlotinib and OS. In light of these findings and the results of the BATTLE trial, cyclin D1 immunohistochemical staining appears to be a promising biomarker for predicting erlotinib sensitivity and additional clinical testing is warranted.

## Figures and Tables

**Table I.** Baseline characteristics.

Characteristic	No. (N=45)	%
Sex		
Female	20	44
Male	25	56
Age (years)		
Median	60	
Range	33–80	
Performance status		
0	8	18
1	37	82
Race/ethnicity		
White	35	78
Black or African American	9	20
Hispanic or Latino	1	2
Pathologic subtype		
Adenocarcinoma	27	60
Squamous cell carcinoma	7	16
Other	11	24
High-risk metastatic sites		
Brain metastases	16	36
Subcutaneous tissue metastases	4	9
Smoking status		
Never	7	16
Distant former (>1 year since cessation)	16	36
Former (1 year - 1 month since cessation)	6	13
Recent former (<1 month since cessation)	8	18
Current	8	18

**Table II.** Adverse events.

Adverse event	Dose-dense chemotherapy						Maintenance Dose-intense erlotinib					
	
	All Grades		Grade 3		Grade 4		Grade 2		Grade 3		Grade 4	
					
	No.	%	No.	%	No.	%	No.	%	No.	%	No.	%
Nausea	28	62	1	2								
Diarrhea	14	31	3	7			19	42	4	9		
Constipation	3	7	1	2								
Anorexia	11	24					2	4	2	4		
Dehydration	8	18	4	9	2	4	1	2	1	2		
Rash	9	20	6	13			6	13	11	24		
Fatigue	27	60	7	16					1	2		
Paronychia							2	4				
Conjunctivis	4	9	2	4								
Mucositis	5	11	1	2					1	2		
Ototoxicity	6	13	3	7								
Peripheral neuropathy	8	18	1	2								
Allergic reaction	3	7	3	7								
Neutropenia	3	7	1	2	1	2						
Hyperbilirubinemia							1	2				
Transaminitis											1	2

**Table III.** EGFR mutation positive cases.

	EGFR mutation testing						
Case	Initial testing	Confirmatory testing	T790M testing	Cyclin D1 (%)	Cyclin D3 (%)	TTP	OS
1	G719X	wt	Negative	Low (25)	High (35)	3.7	10.4
2	Exon 19 del	Exon 19 del	Negative	Low (1)	High (40)	6.3	8.1
3	L858R	L858R	Negative	High (40)	High (40)	49.7	55+

**Table IV.** Characteristics of low versus high cyclin D1 expressing cancers.

Characteristic	Low cyclin D1		High cyclin D1		P-value
	
	No.	%	No.	%	
Sex					0.05
Female	5	29	9	53	
Male	12	71	8	47	
Age (years)					0.98
Median	61		60		
Range	38–72		45–80		
Performance status					0.11
0	2	12	5	29	
1	15	88	12	71	
Race/ethnicity					0.22
White	16	94	13	76	
Black or African American	1	6	3	18	
Hispanic or Latino			1	6	
Pathologic subtype					0.03
Adenocarcinoma	9	53	12	71	
Squamous cell carcinoma	5	29	0	0	
Other	3	18	5	29	
High-risk metastatic sites					0.02
Brain metastases	4	24	8	47	
Subcutaneous tissue metastases	0	0	3	18	
No high-risk sites	13	76	8	47	
Smoking status					0.27
Never	1	6	4	24	
Distant former (>1 year since cessation)	6	35	5	29	
Former (1 year - 1 month since cessation)	3	18	2	12	
Recent former (<1 month since cessation)	4	24	2	12	
Current	3	18	4	24	
Chemotherapy response (N=16 and N=15)					0.51
Progressive disease	3	19	4	27	
Stable disease	9	56	9	60	
Partial response	4	25	2	13	
